# *Caenorhabditis elegans* Exhibits Selective Chemotaxis to Cancer Cell–Conditioned Media

**DOI:** 10.17912/micropub.biology.001618

**Published:** 2025-06-05

**Authors:** Ridvan Aziz Ayaz, Deniz Yozlu, Elif Damla Arisan

**Affiliations:** 1 Institute of Biotechnology, Gebze Technical University, Gebze, Kocaeli, Türkiye; 2 Institute of Neurological Sciences, Department of Neurogenetics, Istanbul University-Cerrahpaşa, Istanbul, Türkiye

## Abstract

*Caenorhabditis elegans* is emerging as a valuable model for investigating chemosensory responses to disease-associated molecular cues recently. In this study, we examined the chemotaxis behavior of *C. elegans* toward the conditioned media from cancerous MIA PaCa-2 and PANC-1 cells and non-cancerous PNT1A cells. Untrained wild-type worms exhibited orientation to PNT1A media compared to the controls. Most importantly, training with cancer-conditioned media led to altered chemotaxis behavior, indicating olfactory learning. These findings support the use of *C. elegans* as a sensitive and adaptable system for detecting cancer-associated metabolites and demonstrate its potential role in non-invasive cancer screening applications and training-based models.

**Figure 1. Training workflow and chemotaxis indices of untrained and trained C. elegans to cancer cell line–conditioned media and cell culture components:**
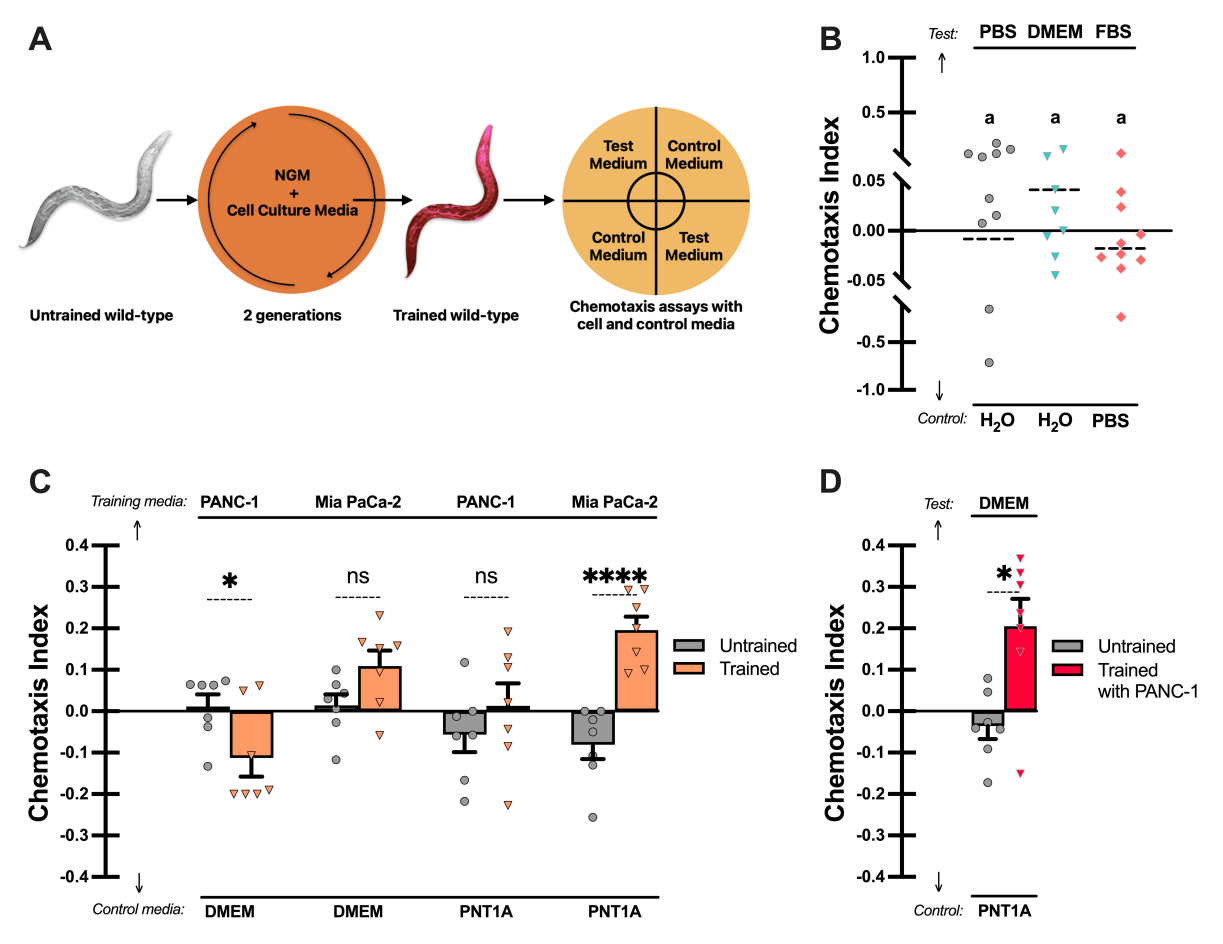
**(A)** Schematic illustration of the training and testing. Wild-type *C. elegans* were grown for two generations on NGM plates supplemented with conditioned media from PANC-1 or Mia PaCa-2 cell lines for training. Trained and untrained worms were then subjected to chemotaxis assays comparing cell and mock-conditioned media. **(B)** Baseline chemotaxis responses of untrained worms to general cell culture-related components (PBS, DMEM, FBS, and H _2_ O). Dashed lines indicate mean, and groups sharing the same letter are not significantly different (compact letter display). **(C)** Chemotaxis indices of untrained (gray) and trained (orange) worms in response to controls versus cancer cell–conditioned media used for training. Each group represents a different pair, as indicated. **(D)** Chemotactic behavior of PANC-1 media-trained worms (red) compared to untrained worms, tested against non-cancerous PNT1A and DMEM as controls. Tukey's test for statistical significance: ns: *p* ≥ 0.05, *: *p* < 0.05, ****: *p* < 0.0001. From **(B)** to **(D)** , positive chemotaxis indices show preference toward upper media (Test or Training) while negative chemotaxis index shows preference of lower media (Control). Error bars in **(C)** and **(D)** show mean ± SEM chemotaxis indices. Individual dots represent technical replicates (n≥7). In all experiments, the population per group ranged from 25 to 300 animals.

## Description

*Caenorhabditis elegans* is a free-living nematode and a widely used model for neurobiology and chemosensation (Leung et al., 2008). It can detect a broad range of volatile and water-soluble compounds via amphid sensory neurons expressing G protein-coupled receptors (GPCRs), enabling responses to trace concentrations of alcohols, ketones, aldehydes, esters, and aromatic compounds (Bargmann, 2006). Distinct sensory neurons such as AWA, AWB, and AWC detect specific chemical classes, and signals are processed by a compact neural network to support navigation through chemical gradients (Bargmann et al., 1993; Troemel et al., 1997). In addition to innate responses, *C. elegans* exhibits robust olfactory learning and memory (Ardiel and Rankin, 2010). Conditioning also enhances sensitivity to subtle differences in odor profiles (Wes and Bargmann, 2001; Worthy et al., 2018). This learning capacity and sensitive chemosensation has led to biosensing applications including disease diagnostics like cancer (Alsaleh et al., 2021; Backes et al., 2021; di Luccio et al., 2022).

Cancer is a multifactorial disease involving abnormal growth, immune evasion, and metastasis and often results with tumors (Brown et al., 2023). These tumors release various metabolites into surrounding fluids, many of which are present at low concentrations and require moderate to advanced analytical methods to detect (García-Cañaveras and Lahoz, 2021; Wang et al., 2023). Conventional screening techniques for such metabolites are often invasive or targeted (Kettritz, 2011; Ma et al., 2024). As a non-invasive alternative, the chemotactic behavior of *C. elegans* has been used to detect cancer-specific odours in human urine (Hirotsu et al., 2015; Lanza et al., 2021). Rather than identifying individual compounds, this approach relies on the recognition of disease-specific odorant signatures, making it cost-effective and accessible.

Based on these, we evaluated whether *C. elegans* can detect cancer-associated volatiles in conditioned media from cultured cell lines, thereby extending prior work beyond patient urine. Phosphate-buffered saline (PBS), Dulbecco's Modified Eagle Medium (DMEM), and fetal bovine serum (FBS) are standard constituents of *in vitro* cell culture systems, each containing defined concentrations of salts, amino acids, and other bioactive molecules. These components were first evaluated to assess whether commonly used media components, either individually or in combination, elicit baseline chemotaxis responses independently of cancer cell-derived volatiles. No significant differences in chemotaxis index were found between the groups ( Figure 1B ), indicating that these media do not elicit differentiable baseline attraction. We next tested whether prior exposure enhances detection capacity of cancer-related cues in *C. elegans* . Conditioned media from PANC-1 and Mia PaCa-2 (pancreatic cancer) were collected from near-confluent cultures, filtered, and incorporated into nematode growth media. Worms were grown and trained on these plates for two generations. Then, chemotaxis assays compared the responses of trained and untrained worms to cancer-conditioned versus cell-free mock-conditioned control media (illustrated in Figure 1A ). Within untrained populations, worms were slightly attracted to PNT1A media rather than PANC-1 or Mia PaCa-2 media ( Figure 1C, PNT1A control media groups). Same result was also seen in mock-conditioned DMEM versus PNT1A group with the untrained worms ( Figure 1D ). In the DMEM (mock) versus PANC-1 or Mia PaCa-2 plates, untrained worms exhibited no noticeable preference ( Figure 1C ). This finding indicates that untrained worms were actually attracted to PNT1A conditioned-media compared to cancerous cell media or mock media. When worms were trained with PANC-1 media and placed on the PANC-1 vs. DMEM plates, worms showed avoidance of PANC-1 and preference toward the mock DMEM, resulting in a significant difference in chemotaxis behavior compared to the untrained group. When PANC-1 trained worms were assayed for PANC-1 versus PNT1A, no apparent preference was noticeable, and no significant differences in chemotaxis indices were observed between this trained and the untrained population ( Figure 1C ). Mia PaCa-2 trained worms displayed attraction to Mia PaCa-2 in DMEM and PNT1A-controlled groups, but difference were not statistically significant in the case of DMEM ( *p* =0.1128). Importantly, while training with PANC-1 media results in aversion from PANC-1 media, training with Mia PaCa-2 media exhibited enhanced attraction to Mia PaCa-2 ( Figure 1C ). While both cell lines originate from pancreatic cancer, a contrast in chemotaxis behavior was noticeable between the assays.

To test discriminatory learning and the avoidance effect of PNT1A on the trained worms, we assessed untrained and PANC-1 media-trained worms in assays comparing PNT1A-conditioned and mock-conditioned DMEM. Trained worms showed preference toward DMEM, while untrained worms slightly preferring PNT1A, resulting in a significant difference upon training ( Figure 1D ). This shift indicates that training on cancer cues alters olfactory expectations and supports learned discrimination. Additionally, PNT1A media caused mild attraction in untrained worms but was avoided by cancerous cell media-trained worms ( Figure 1C and 1D). While comprehensive volatile organic compounds or metabolite profiles comparing PNT1A with cancer cells are limited, it is established that cell type and metabolic states affect volatile emissions (Filipiak et al., 2016). Therefore, our findings suggest that not only does PNT1A have a unique volatile signature and is distinguishable from cancer cells, but PANC-1 and Mia PaCa-2 can also be individually differentiated by *C. elegans* after training. Additionally, the mild baseline attraction to PNT1A and subsequent avoidance following training imply that *C. elegans* perceives and processes these profiles differently after exposure and possible transgenerational associative learning.

The observed chemotaxis behaviors also correspond with known cancer-associated compounds. Both PANC-1 and Mia PaCa-2 release aldehydes, ketones, and fatty acid derivatives such as 2-nonanone and 2-pentanone (Daulton et al., 2021) which are established ligands for *C. elegans* chemosensory receptors (Bargmann, 2006). Although both cell lines originate from pancreatic tumors, they vary in differentiation status, metabolic activity, and volatile emission profiles (Filipiak et al., 2016; Daulton et al., 2021). These differences likely underlie the distinct and opposite chemotactic responses we observed following training as aversion to PANC-1 and mild attraction to Mia PaCa-2 ( Figure 1C ). Additionally, prior studies by Hirotsu et al. (2015) have also demonstrated strong responses to cancer-conditioned media, reinforcing our conclusion that *C. elegans* can detect cancer-specific volatiles across various cell types.

Our results and the methodology show that *C. elegans* can distinguish between cancerous and non-cancerous volatiles following associative training in possible *ex vivo* conditions. Unlike urine-based platforms, our approach utilizes cell culture-derived media for both training and testing, enabling controlled experiments and facilitating potential integration with metabolomic analyses. That said, since this study is based on controlled chemotaxis assays, further analytical studies and direct assessments of volatile profiles from additional, comparable cancer cell lines are necessary. Therefore, this preliminary study and the workflow model can serve as an accessible educational tool for further *C. elegans* training, while also highlighting the importance of metabolic and microenvironmental context in cancer profiles.

## Methods

**Strain Maintenance and Synchronization :** All experiments were conducted using the wild-type *Caenorhabditis elegans* N2 strain. Worms were maintained on NGM+cholesterol plates seeded with *Escherichia coli* OP50 and stored at 4°C until use. Prior to transferring worms, fresh OP50 was seeded onto NGM plates and incubated overnight at 37°C to allow bacterial growth. N2 *C. elegans* and *E. coli* OP50 were provided by the CGC, which is funded by NIH Office of Research Infrastructure Programs (P40 OD010440). Synchronization was performed via standard bleaching. Gravid adults were collected and treated with a bleach solution to isolate embryos, which were then incubated overnight in M9 buffer at 20°C with shaking to allow hatching. The resulting L1 larvae were then allowed to grow until L4 to be used for subsequent experiments.

**Chemotaxis Assays** : All chemotaxis assays were carried out on 40 mm NGM plates without bacterial lawn. L4-stage worms were collected in S-buffer and washed several times via centrifugation to remove residual bacteria. Test and control solutions (4 μL each) with sodium azide (10%) were spotted onto designated quadrants of the assay plates ( Figure 1A ), and approximately 25–300 worms were placed in the center of each plate. After one hour at room temperature, the number of worms in the test and control areas was counted, and the chemotaxis index was calculated as Chemotaxis Index = (Number at Test – Number at Control) / (Total Number at Test and Control), ranging from -1 to +1, with positive values indicating attraction toward the tested media and negative values indicating avoidance. Finished assay plates were stored at 4°C until counting if immediate counting was not possible. Chemotaxis responses to phosphate-buffered saline (PBS), fetal bovine serum (FBS), Dulbecco's Modified Eagle Medium (DMEM), and distilled water (H _2_ O) were also tested to evaluate baseline detections.

**Response to Cancer Cell Culture Supernatants :** Conditioned media from cancer cell lines (MIA PaCa-2, PANC-1) and a non-cancerous prostate epithelial cell line (PNT1A) were tested. Media from these cell cultures were collected at full confluency, filtrated (0.22 μm), and stored at −20°C with constant sterility. Sterile DMEM was also incubated with same conditions and durations as the cells (37°C, 5% CO _2_ ) and used as the mock-conditioned control in cell-media conditioned tests in order to account for nonspecific sensory adaptation or media-related effects. The pH of each media were tested prior to use.

**Training Worms with Cancer-Associated Cues** : To evaluate learning and memory capabilities, worms were trained by growing on plates seeded with UV-killed OP50 and conditioned media from cancer cell lines. For this, previously obtained eggs were placed in training plates and allowed to grow until gravid adults are obtained. These adults then collected and bleached to obtain the new eggs. These eggs then reintroduced to another identical training plates. After at least two generations on these plates, L4 synchronized progeny were tested in chemotaxis assays to assess whether prior exposure influenced their response to cancer related cues.

**Statistical Analysis** : All statistical analyses were performed using GraphPad Prism 10 for macOS (GraphPad Software, Boston, Massachusetts USA). For Figure 1B and Figure 1C, group differences were analyzed using one-way and two-way ANOVA, respectively, followed by Tukey's multiple comparisons test. For Figure 1D, unpaired t-tests with Welch's correction were used for pairwise comparisons. Normality of data was assessed to confirm suitability for parametric testing. Results were considered statistically significant at *p* < 0.05.
